# Supplementary material for: A multi-classifier system integrated by clinico-histology-genomic analysis for predicting recurrence of papillary renal cell carcinoma
Source: Nat Commun. 2024 Jul 23;15:6215. doi: 10.1038/s41467-024-50369-y (PMC11266571; doi:10.1038/s41467-024-50369-y)
Supplement: Supplementary file 1 — Supplementary Information [file 41467_2024_50369_MOESM1_ESM.pdf]

Supplementary Information

A multi-classifier system integrated by clinico-histology-genomic analysis for predicting recurrence of papillary renal cell carcinoma

Huang et al.

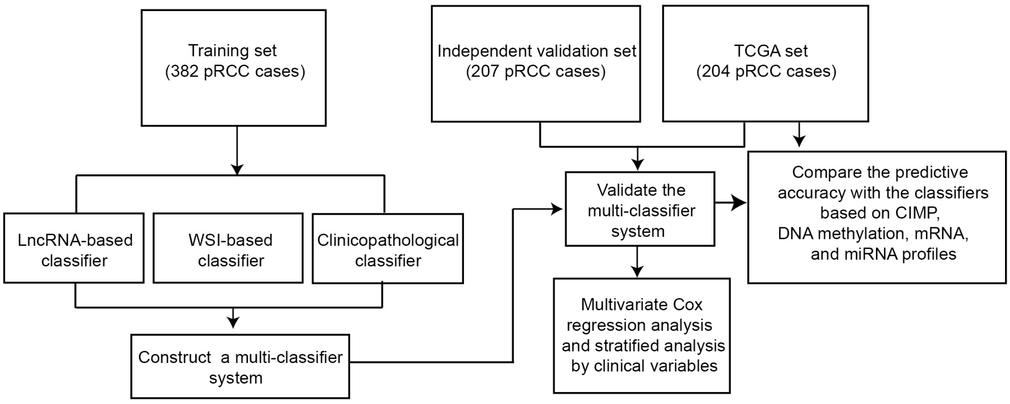

Supplementary Fig. 1 Study design. pRCC = papillary renal cell carcinoma; TCGA = The Cancer Genome Atlas; CIMP = CpG island methylator phenotype.

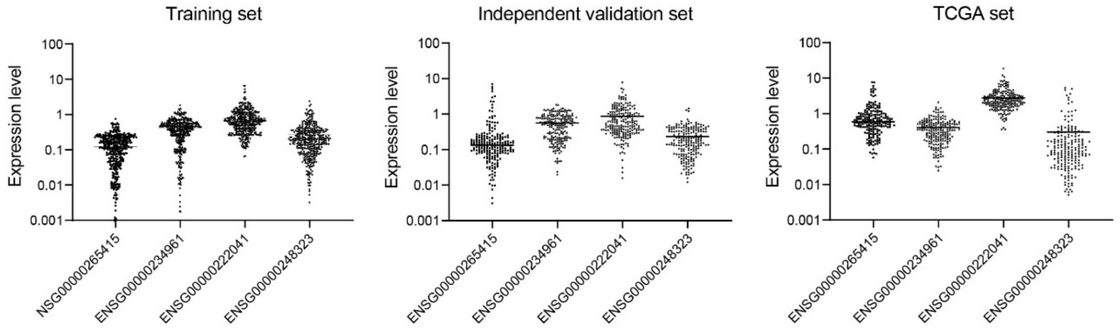

Supplementary Fig. 2 Scatter dot plot depicting the expression of the four lncRNAs in the three different sets. The expression levels of the four lncRNAs in the training set and independent validation set were represented by $2^{-\Delta Ct}$. For the TCGA set, lncRNA levels were represented by RPKM. Source data are provided as a Source Data file.

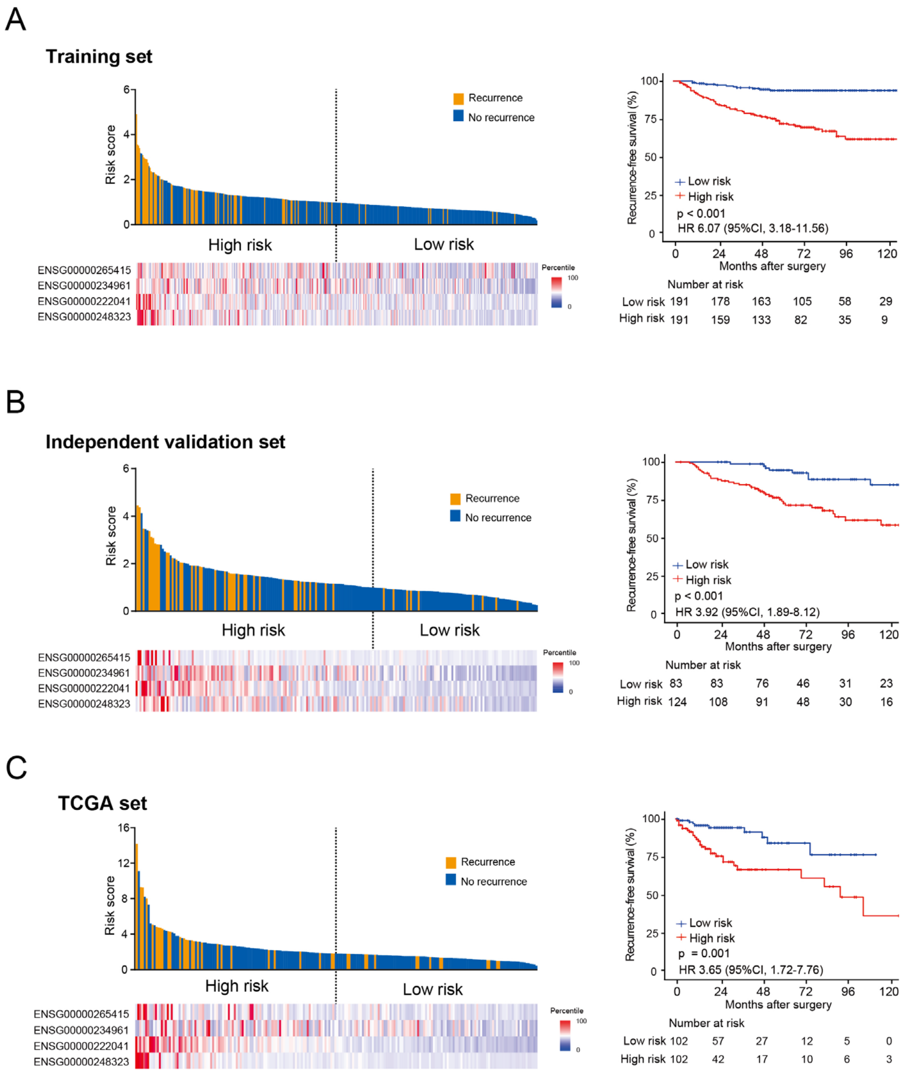

Supplementary Fig. 3 The risk score calculated by the lncRNA-based classifier and Kaplan-Meier survival for RFS in the three different sets. (A) The risk score calculated by the lncRNA-based classifier and Kaplan-Meier survival in the training set. Upper left of panel: risk-score distribution of the lncRNA-based classifier and patient progression status. Patients in the training set were divided into high-risk and low-risk groups, with the median risk score (0.9800) as the cutoff. Lower left of panel: heatmap shows the expression level of four lncRNAs. Right of panel: Kaplan-Meier survival analysis for RFS in pRCC patients who were divided into low-risk and high-risk groups according to four-lncRNA-based risk score. **(B, C)** illustrates the risk score calculated by the lncRNA-based classifier and Kaplan-Meier survival in the independent validation set and TCGA set, respectively. Risk score for each patient in the independent validation set and the TCGA set was calculated with the same formula used in the Training set. Patients in the independent validation set were classified into high-risk and low-risk groups with the same cutoff used in the Training set. Patients in the TCGA set were divided into high-risk and low-risk groups, using the median risk score (1.8100) as the cutoff. P-value were calculated with log-rank test. HR and 95% CI were calculated using the Cox proportional-hazards model. Source data are provided as a Source Data file.

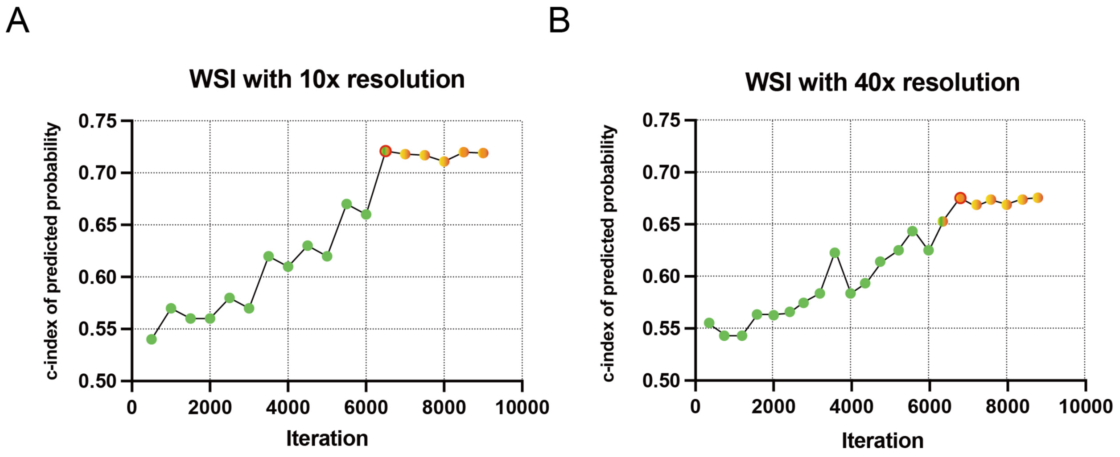

Supplementary Fig. 4 Selection of the optimal deep learning WSI-based models. (A) The C-indexes of the candidate deep learning WSI-based models with 10× resolution in the tuning set. The red cycle indicates the selected model, yellow points indicate models not selected. The c-indexes of the models from the half of the developing run are shown as green points for comparison. **(B)** The C-indexes of the candidate deep learning WSI-based models with 40× resolution in the tuning set. Source data are provided as a Source Data file.

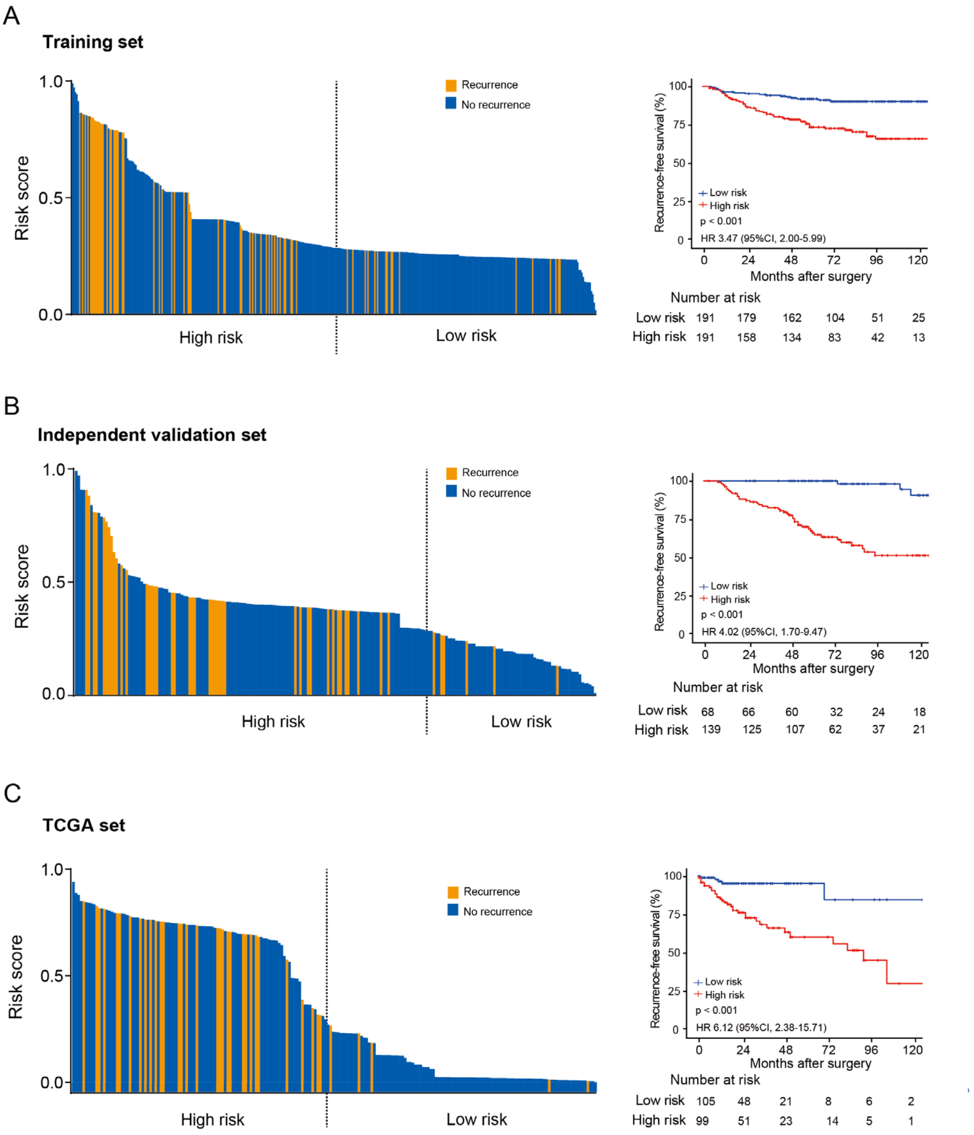

Supplementary Fig. 5 The risk score calculated by the WSI-based classifier and Kaplan-Meier survival for RFS in the three different sets. (A) The risk score calculated by the WSI-based classifier and Kaplan-Meier survival in the training set. Patients in the training set were divided into high-risk and low-risk groups, with the median risk score (0.2857) as the cutoff. Left of panel: risk-score distribution of the WSI-based classifier and patient progression status. Right of panel: Kaplan-Meier survival analysis for RFS in pRCC patients who were divided into low-risk and high-risk groups according to WSI-based risk score. **(B, C)** illustrates the risk score calculated by the WSI-based classifier and Kaplan-Meier survival in the independent validation set and TCGA set, respectively. Risk score for each patient in the independent validation set and the TCGA set was both calculated with the same formula used in the training set. Patients in these two sets were also divided into high-risk and low-risk groups using the same cutoff in the training set. P-value were calculated with log-rank test. HR and 95% CI were calculated using the Cox proportional-hazards model. Source data are provided as a Source Data file.

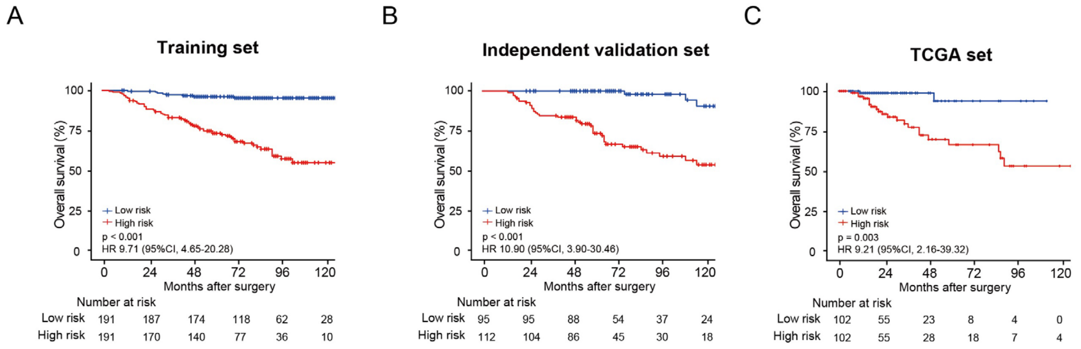

Supplementary Fig. 6 Kaplan-Meier survival analysis for OS of the multi-classifier system in the three sets. Patients with pRCC in the training set (**A**), independent validation set (**B**) and TCGA set (**C**) were divided into low-risk and high-risk groups according to multi-classifier system. P-value, HR and 95% CI were computed using the Cox proportional-hazards model. Source data are provided as a Source Data file.

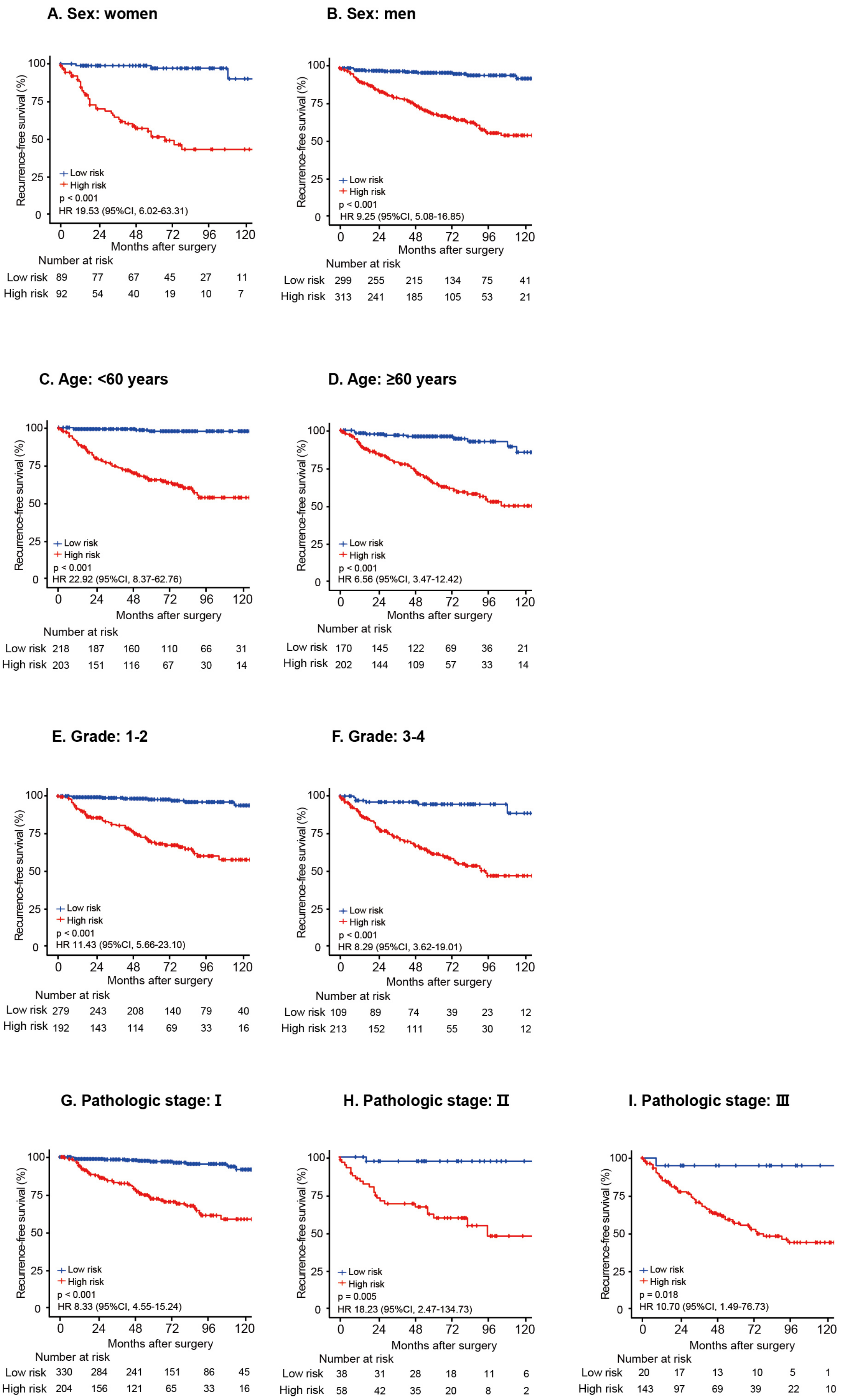
A. Sex: women
Recurrence-free survival (%)
100
75
50
25
0
Low risk
High risk
p < 0.001
HR 19.53 (95%CI, 6.02-63.31)
0 24 48 72 96 120
Months after surgery
Number at risk
Low risk 89 77 67 45 27 11
High risk 92 54 40 19 10 7
B. Sex: men
Recurrence-free survival (%)
Low risk
High risk
p < 0.001
HR 9.25 (95%CI, 5.08-16.85)
0 24 48 72 96 120
Months after surgery
Number at risk
Low risk 299 255 215 134 75 41
High risk 313 241 185 105 53 21
C. Age: <60 years
Recurrence-free survival (%)
Low risk
High risk
p < 0.001
HR 22.92 (95%CI, 8.37-62.76)
Months after surgery
Number at risk
Low risk 218 187 160 110 66 31
High risk 203 151 116 67 30 14
D. Age: ≥60 years
Recurrence-free survival (%)
Low risk
High risk
p < 0.001
HR 6.56 (95%CI, 3.47-12.42)
Months after surgery
Number at risk
Low risk 170 145 122 69 36 21
High risk 202 144 109 57 33 14
E. Grade: 1-2
Recurrence-free survival (%)
Low risk
High risk
p < 0.001
HR 11.43 (95%CI, 5.66-23.10)
Months after surgery
Number at risk
Low risk 279 243 208 140 79 40
High risk 192 143 114 69 33 16
F. Grade: 3-4
Recurrence-free survival (%)
Low risk
High risk
p < 0.001
HR 8.29 (95%CI, 3.62-19.01)
Months after surgery
Number at risk
Low risk 109 89 74 39 23 12
High risk 213 152 111 55 30 12
G. Pathologic stage: I
Recurrence-free survival (%)
Low risk
High risk
p < 0.001
HR 8.33 (95%CI, 4.55-15.24)
Months after surgery
Number at risk
Low risk 330 284 241 151 86 45
High risk 204 156 121 65 33 16
H. Pathologic stage: II
Recurrence-free survival (%)
Low risk
High risk
p = 0.005
HR 18.23 (95%CI, 2.47-134.73)
Months after surgery
Number at risk
Low risk 38 31 28 18 11 6
High risk 58 42 35 20 8 2
I. Pathologic stage: III
Recurrence-free survival (%)
Low risk
High risk
p = 0.018
HR 10.70 (95%CI, 1.49-76.73)
Months after surgery
Number at risk
Low risk 20 17 13 10 5 1
High risk 143 97 69 39 22 10

Supplementary Fig. 7 Kaplan-Meier survival analysis for RFS of the multi-classifier system in different subgroups stratified by clinicopathological risk factors. Sex (**A, B**), age (**C, D**), and grade (**E, F**) and pathologic stage (**G-I**). P value, HR and 95% CI were calculated using the Cox proportional-hazards model. Source data are provided as a Source Data file.

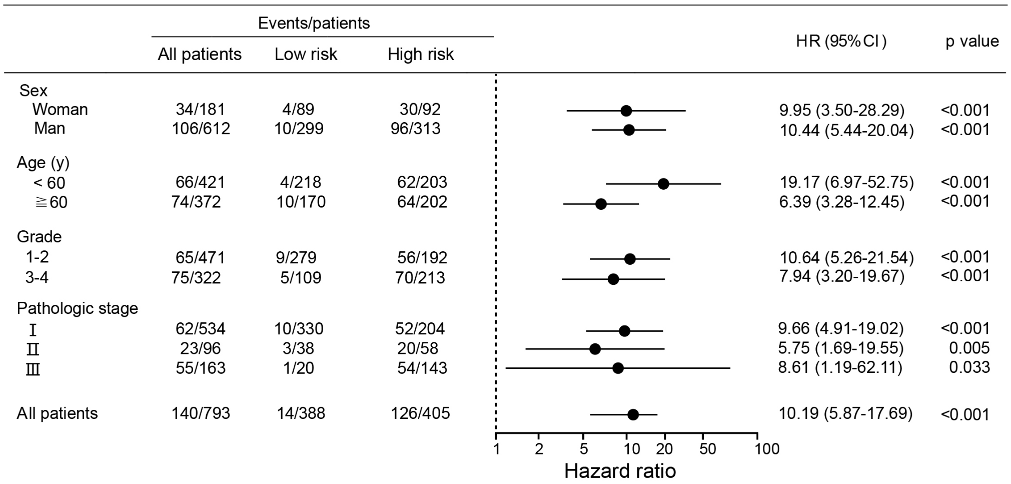

Supplementary Fig. 8 HR of OS for all 941 patients with pRCC according to the multi-classifier system in different groups stratified by clinical parameters. HR and 95% CI were computed using the Cox proportional-hazards model. Source data are provided as a Source Data file.

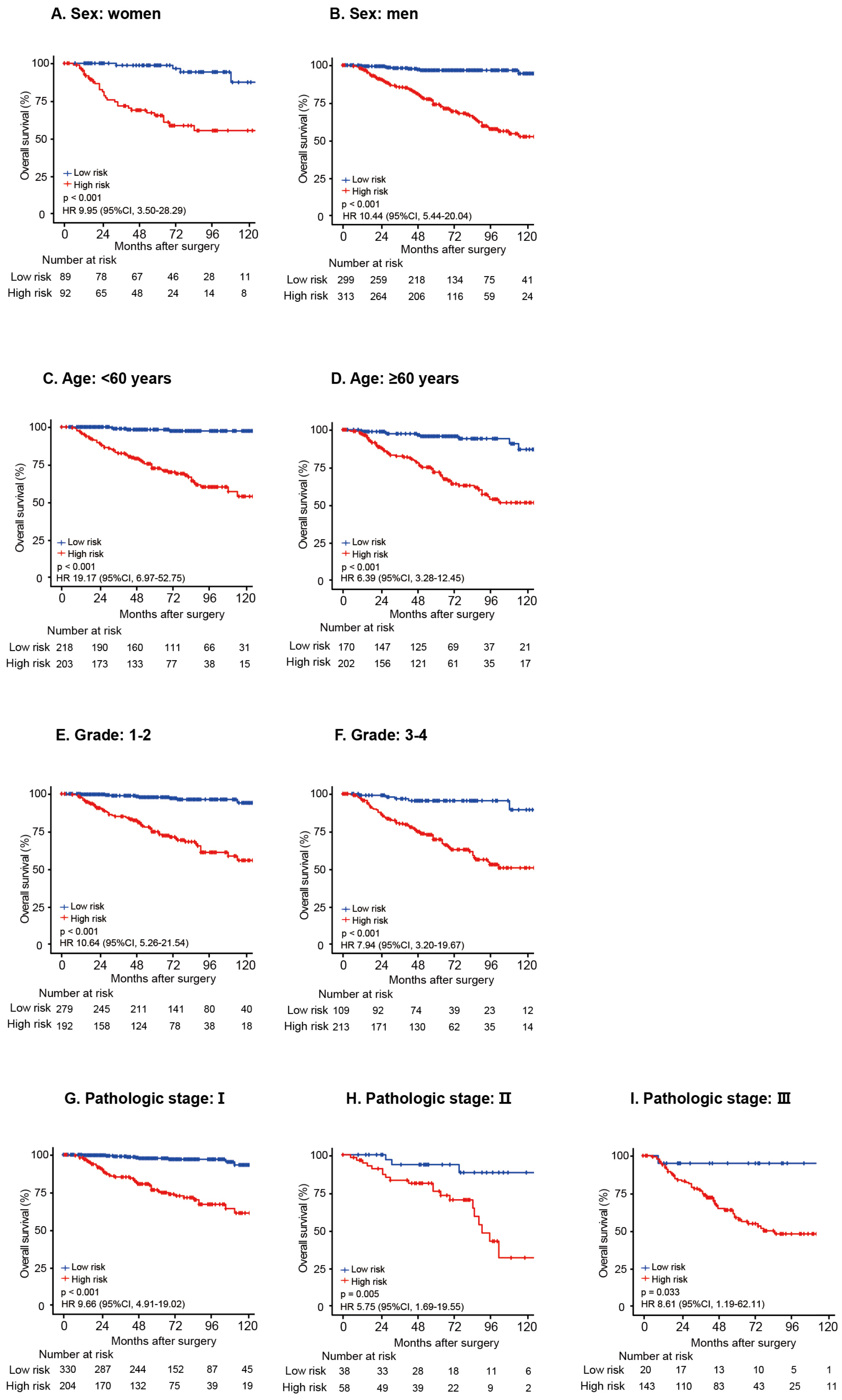
A. Sex: women
Overall survival (%)
Low risk
High risk
p < 0.001
HR 9.95 (95%CI, 3.50-28.29)
Months after surgery
Number at risk
Low risk 89 78 67 46 28 11
High risk 92 65 48 24 14 8
B. Sex: men
Overall survival (%)
Low risk
High risk
p < 0.001
HR 10.44 (95%CI, 5.44-20.04)
Months after surgery
Number at risk
Low risk 299 259 218 134 75 41
High risk 313 264 206 116 59 24
C. Age: <60 years
Overall survival (%)
Low risk
High risk
p < 0.001
HR 19.17 (95%CI, 6.97-52.75)
Months after surgery
Number at risk
Low risk 218 190 160 111 66 31
High risk 203 173 133 77 38 15
D. Age: ≥60 years
Overall survival (%)
Low risk
High risk
p < 0.001
HR 6.39 (95%CI, 3.28-12.45)
Months after surgery
Number at risk
Low risk 170 147 125 69 37 21
High risk 202 156 121 61 35 17
E. Grade: 1-2
Overall survival (%)
Low risk
High risk
p < 0.001
HR 10.64 (95%CI, 5.26-21.54)
Months after surgery
Number at risk
Low risk 279 245 211 141 80 40
High risk 192 158 124 78 38 18
F. Grade: 3-4
Overall survival (%)
Low risk
High risk
p < 0.001
HR 7.94 (95%CI, 3.20-19.67)
Months after surgery
Number at risk
Low risk 109 92 74 39 23 12
High risk 213 171 130 62 35 14
G. Pathologic stage: I
Overall survival (%)
Low risk
High risk
p < 0.001
HR 9.66 (95%CI, 4.91-19.02)
Months after surgery
Number at risk
Low risk 330 287 244 152 87 45
High risk 204 170 132 75 39 19
H. Pathologic stage: II
Overall survival (%)
Low risk
High risk
p = 0.005
HR 5.75 (95%CI, 1.69-19.55)
Months after surgery
Number at risk
Low risk 38 33 28 18 11 6
High risk 58 49 39 22 9 2
I. Pathologic stage: III
Overall survival (%)
Low risk
High risk
p = 0.033
HR 8.61 (95%CI, 1.19-62.11)
Months after surgery
Number at risk
Low risk 20 17 13 10 5 1
High risk 143 110 83 43 25 11

Supplementary Fig. 9 Kaplan-Meier survival analysis for OS of the multi-classifier system in different subgroups stratified by clinicopathological risk factors. Sex (**A, B**), age (**C, D**), and grade (**E, F**) and pathologic stage (**G-I**). P value, HR and 95% CI were calculated using the Cox proportional-hazards model. Source data are provided as a Source Data file.

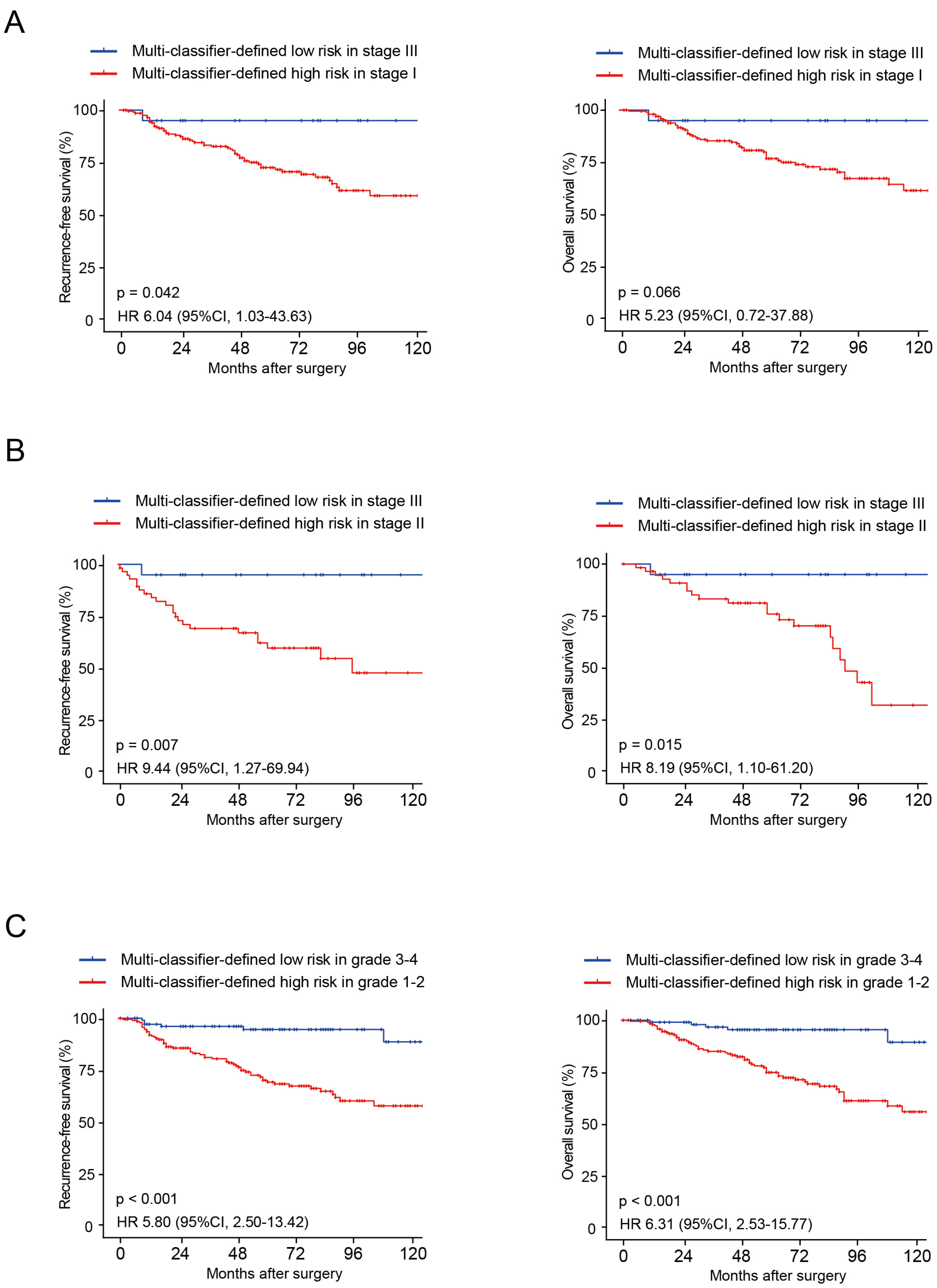

Supplementary Fig. 10 Kaplan–Meier survival analysis in multi-classifier risk-score-defined low-risk and high-risk patients stratified by stage and grade. Kaplan-Meier survival analysis for RFS (left) and OS (right) of the multi-classifier risk score in low-risk stage III patients and high-risk stage I patients (A), low-risk stage III patients and high-risk stage II patients (B), low-risk grade 3-4 patients and high-risk grade 1-2 patients (C). HRs and 95% CIs were calculated using the Cox proportional hazards model. Source data are provided as a Source Data file.

Supplementary Table 1 Information and primers for 40 lncRNAs

ID	Chr	Start	End	Width	Stand	Gene name	Forward Primer (5'-3')	Reverse Primer (5'-3')
ENSG00000162888	chr1	206491116	206497728	6613	-	C1orf147	GTCTGCCACTTACTAGCCAT	ACAACACCTGCCCTATACAGT
ENSG00000172965	chr2	111196350	111495100	298751	-	MIR4435-1HG	AGAGAATGTCGGGAGAGGAAGT	AAAAAGCAGCGACCATCCAGT
ENSG00000177133	chr1	3059615	3068437	8823	-	LINC00982	TGCTCTAGCGCCCACTATTT	ACGAAAGGGGAGAGCAACTT
ENSG00000187621	chr14	95650498	95679833	29336	+	TCL6	AGAGGGGACCATGCAAAGGA	ACCTCCCCAGCTTCTGTTGT
ENSG00000188242	chr5	466124	473098	6975	-	PP7080	AGCCACACAAAAGCCTGCAT	AGGGCTTGCTGCATAGAGGA
ENSG00000203999	chr20	50292720	50314922	22203	+	LINC01270	CTAACTTGCCTCGAAGACCA	GACACCTCATTCATATGTGCT
ENSG00000213373	chr17	42874670	42898704	24035	-	LINC00671	GCACCCCCAGGAAGTTTACC	CAAGGTGGCCTGTTGGAAGG
ENSG00000214922	chr6	29726601	29749049	22449	-	HLA-F-AS1	ATCAAGTTTCCGCTGACCAC	AGCCAGTCGAACATATGCC
ENSG00000216560	chr4	3576869	3590711	13843	+	LINC00955	AGTGAGGGTCCGAGGGATTC	GCCCACAACCAGCTCAAGAA
ENSG00000222041	chr2	87455368	87606805	151438	+	CYTOR	TCGAATATGACAGACACCGAA	ACAAATGGGAAACCGACCAG
ENSG00000227230	chr1	243135898	243140588	4691	+	RP11-261C10.5	ACCAGATCGACCGTTGTTTCC	CTCATAAAGGTTTGCTTTCGTCCC
ENSG00000229005	chr20	44372746	44395706	22961	-	HNF4A-AS1	CCCAACCACTGACCAAACTCC	CTGCCGTTGGTATCAGCAAGT
ENSG00000229953	chr1	156646507	156661424	14918	-	RP11-284F21.7	GGCTGGAACTTAACGCTGTC	GACTCTGAGAGCAGGGCAAG
ENSG00000231310	chr3	177037405	177047923	10519	+	TBL1XR1-AS1	TGACAGTGATTGCTCTGGACC	TTTGATGTTCTTCCCAGTCCACC
ENSG00000231868	chr1	6443034	6447006	3973	-	RP1-202O8.2	TGGGAGCTTGGTCTAAGGTGG	TCGGCAAGTTGTTTCCAGGTG
ENSG00000233154	chr1	116423724	116478842	55119	-	RP4-655J12.4	TTCCTGTGGGGTCTGACAGT	GCCATTGCAGATGACCTCGT
ENSG00000234961	chr10	17233325	17234833	1509	-	lnc-TRDMT1-5	TCAAGGTCAAGACGTGCCAG	AGGGTCATAAAATGTGTCAACGG
ENSG00000236472	chr17	50135586	50146176	10591	+	AC002401.1	CTCCGACTCCTCCAGAACCC	TTGATCGTTGTTCTTGTGCAT
ENSG00000241224	chr3	109118252	109150514	32263	+	FLJ22763	CTTTTACTCTCTATTGCCTC	AGCCACTATCTACTTGTTG
ENSG00000248323	chr5	91303029	91314402	11374	-	LUCAT1	AAACCATGTGTCAAGCTCGGAT	TGCCAAGGTCCCATAAGAGT
ENSG00000249835	chr5	83531352	83581320	49969	-	VCAN-AS1	AGAGCTAATGCCACATCACAGC	AGCCACCAACATACTTGACAGA
ENSG00000251165	chr4	186286094	186500997	214904	-	F11-AS1	TTCGCCGTGTTACCTTCTCA	GGGAATGAAGCACAACAGGACA
ENSG00000251194	chr11	35212550	35214007	1458	+	RP1-68D18.2	CACATGCTTGGCCTCATTTC	GTGTAAGGGGCTTTGTCTTGC
ENSG00000251442	chr4	78645903	78682699	36797	+	LINC01094	TTGTTTGGCAGGCACTCCAT	TGTTGTCTCACCACCAGCAG
ENSG00000255443	chr11	35210343	35214985	4643	-	RP1-68D18.4	ACTTCGTTGGTAGTCATCATTAGGC	CCAAGACACATTCCACCCCAG
ENSG00000257027	chr12	9658567	9662085	3519	+	RP11-705C15.3	GATGAGACTTAGCGTGCCTGT	TCCTCGGGTTGCCTCTGTTTA
ENSG00000257139	chr12	70180338	70202004	21667	+	RP11-320P7.2	GGGACTTAATCCAGCATCCCCTT	TGAGAAATTCCATGATACGCCAT
ENSG00000258017	chr12	49127782	49147869	20088	+	RP11-386G11.10	ACAAAATAGAACATGGAC	CACCTAGACTCTATCTTC
ENSG00000261420	chr6	166383189	166384824	1636	+	RP1-168L15.5	GATCATTAGCACGCGACCCTC	TGATTACAGGCGTCAGTCAC
ENSG00000261438	chr10	89015836	89017059	1224	+	RP11-399O19.9	TTCTTTCCTGCATATTATCCA	AACTATCTTACCGACATGCTT
ENSG00000261795	chr7	161765	164972	3208	-	RP11-90P13.1	GTCCAGGCTCTAAGCAAACG	GTCGGAGAAGCACTCAGGTC
ENSG00000265415	chr17	59202677	59203829	1153	-	AC099850.3	GGTCAGCACAAACCGTAAACAGC	CCTCCCCACCCCTTCGTCT
ENSG00000269974	chr15	30648797	30649529	733	+	RP11-932O9.10	GTGCCCCCTGTTGACATTTTC	TCACAGACTACGTTAAGGACCC
ENSG00000271590	chr2	111210995	111212476	1482	-	RP11-181E10.3	CATGTTATACCCTTGAATGGC	CCAAGGGGATACTAGAACCA
ENSG00000273486	chr3	136837338	136839021	1684	-	RP11-731C17.2	GAACACCCCAGGATCAACAGA	TGGCCTCAAGAAGGAGTTCAG
ENSG00000276980	chr19	6716386	6717742	1357	-	CTD-3128G10.7	AATTGCTTCTCCCATCGCCTC	CTCCTCCCATCCTTCTCGCTTC
ENSG00000278898	chr8	134764809	134767276	2468	+	CTD-2342N23.1	TGAGGCAAACAACCTCCTGG	TGGAAGCAGACACGCATCAA
ENSG00000279191	chr2	111321433	111324812	3380	-	RP11-803D5.1	TGGCAAGGGGAATCTGTCAT	CACAGGTGAAGGAAGGGGAT
ENSG00000280055	chr8	127946559	127948723	2165	-	TMEM75	ACCTGAGGCCCACCTTGTAA	ACAAGCCGTTGAAAGTCCCC
ENSG00000280384	chr22	46163303	46165347	2045	+	RP4-695O20.1	ACACTGCCTGCTTAGTTGTGG	GCCTGCTCTTCCCGCTTATG

Supplementary Table 2 Univariate association of 40 lncRNAs with RFS in the Training set

ID	HR	95%CI		p value
		Lower	Upper	
ENSG00000162888	0.918	0.715	1.177	0.499
ENSG00000172965	0.900	0.736	1.100	0.303
ENSG00000177133	0.993	0.873	1.128	0.909
ENSG00000187621	0.970	0.819	1.148	0.720
ENSG00000188242	0.855	0.655	1.116	0.249
ENSG00000203999	1.121	0.903	1.391	0.301
ENSG00000213373	0.899	0.670	1.207	0.479
ENSG00000214922	1.050	0.984	1.121	0.137
ENSG00000216560	1.059	0.864	1.299	0.579
ENSG00000222041	1.371	1.240	1.516	<0.001
ENSG00000227230	1.006	0.943	1.073	0.859
ENSG00000229005	1.114	0.844	1.472	0.446
ENSG00000229953	0.965	0.801	1.163	0.708
ENSG00000231310	0.973	0.781	1.212	0.808
ENSG00000231868	0.925	0.777	1.102	0.384
ENSG00000233154	0.999	0.984	1.014	0.860
ENSG00000234961	1.578	1.237	2.012	0.002
ENSG00000236472	1.038	0.985	1.094	0.168
ENSG00000241224	0.995	0.981	1.010	0.501
ENSG00000248323	1.448	1.304	1.607	<0.001
ENSG00000249835	0.964	0.849	1.094	0.570
ENSG00000251165	1.111	0.932	1.324	0.242
ENSG00000251194	0.974	0.859	1.106	0.688
ENSG00000251442	0.852	0.635	1.143	0.284
ENSG00000255443	0.886	0.632	1.242	0.483
ENSG00000257027	1.080	0.893	1.307	0.428
ENSG00000257139	0.911	0.691	1.201	0.508
ENSG00000258017	0.914	0.754	1.108	0.360
ENSG00000261420	0.895	0.688	1.165	0.410
ENSG00000261438	1.007	0.788	1.288	0.956
ENSG00000261795	0.971	0.720	1.308	0.845
ENSG00000265415	1.486	1.112	1.985	0.007
ENSG00000269974	1.085	0.967	1.217	0.166
ENSG00000271590	0.931	0.782	1.109	0.423
ENSG00000273486	0.848	0.645	1.115	0.238
ENSG00000276980	0.992	0.737	1.335	0.955
ENSG00000278898	0.893	0.658	1.212	0.468
ENSG00000279191	0.982	0.927	1.040	0.528
ENSG00000280055	0.952	0.731	1.239	0.713
ENSG00000280384	0.842	0.614	1.153	0.283

HRs, 95% CIs and two-sided P values were calculated using the Cox proportional hazards model. Source data are provided as a Source Data file.

Supplementary Table 3 Univariate and multivariate Cox regression analysis of clinicopathological factors with RFS in the training set

	Univariate analysis		Multivariate analysis	
	**HR (95%CI)**	**p value**	**HR (95%CI)**	**p value**
Sex (woman vs man)	1.14 (0.66-1.97)	0.640	1.10 (0.63-1.91)	0.741
Age (≧60 years vs < 60 years)	1.32 (0.82-2.11)	0.256	1.40 (0.87-2.26)	0.168
Grade (4 vs 3 vs 2 vs 1)	1.98 (1.34-2.92)	<0.001	1.76 (1.17-2.63)	0.006
Pathologic stage (III vs II vs I)	3.26 (1.95-5.47)	<0.001	2.98 (1.76-5.04)	<0.001

HR=hazard ratio; CI=confidence interval; WHO=World Health Organization; ISUP=International Society of Urological Pathology. HRs, 95% CIs and two-sided P values were calculated using the Cox proportional hazards model. Source data are provided as a Source Data file.

Supplementary Table 4 Univariate Cox regression analysis of the multi-classifier system with RFS in the three sets

	Training set (n=382)		Independent validation set (n=207)		TCGA set (n=204)	
	**HR (95%CI)**	**p value**	**HR (95% CI)**	**p value**	**HR (95% CI)**	**p value**
Sex (woman vs man)	1.14 (0.66-1.97)	0.640	1.52 (0.81-2.83)	0.189	2.05 (1.02-4.12)	0.044
Age (≧60 years vs < 60 years)	1.32 (0.82-2.11)	0.256	1.44 (0.81-2.55)	0.211	0.83 (0.43-1.59)	0.571
Grade (4 vs 3 vs 2 vs 1)	1.98 (1.34-2.92)	<0.001	2.08 (1.30-3.35)	0.002	2.04 (1.14-3.65)	0.016
Pathologic stage (III vs II vs I)	3.26 (1.95-5.47)	<0.001	2.66 (1.41-5.01)	0.003	6.90 (3.36-14.18)	<0.001
Multi-classifier system (High risk vs Low risk)	11.17 (5.11-24.40)	<0.001	12.85 (4.61-35.84)	<0.001	8.54 (3.02-24.14)	<0.001

TCGA=The Cancer Genome Atlas; HR=hazard ratio; CI=confidence interval. HRs, 95% CIs and two-sided P values were calculated using the Cox proportional hazards model. Source data are provided as a Source Data file.

Supplementary Table 5 Univariate Cox regression analysis of the multi-classifier system with OS in the three sets

	Training set (n=382)		Independent validation set (n=207)		TCGA set (n=204)	
	**HR (95%CI)**	**p value**	**HR (95% CI)**	**p value**	**HR (95% CI)**	**p value**
Sex (woman vs man)	1.01 (0.58-1.77)	0.964	1.22 (0.63-2.37)	0.552	1.55 (0.61-3.95)	0.359
Age (≧60 years vs < 60 years)	1.39 (0.87-2.21)	0.170	1.63 (0.90-2.93)	0.106	1.01 (0.44-2.31)	0.988
Grade (4 vs 3 vs 2 vs 1)	2.80 (1.27-6.19)	0.011	3.88 (1.33-11.29)	0.013	3.46 (0.78-15.24)	0.101
Pathologic stage (III vs II vs I)	3.27 (1.96-5.44)	<0.001	2.22 (1.16-4.28)	0.017	8.40 (3.25-21.74)	<0.001
Multi-classifier system (High risk vs Low risk)	9.71 (4.65-20.28)	<0.001	10.90 (3.90-30.46)	<0.001	9.21 (2.16-39.32)	0.003

TCGA=The Cancer Genome Atlas; HR=hazard ratio; CI=confidence interval. HRs, 95% CIs and two-sided P values were calculated using the Cox proportional hazards model. Source data are provided as a Source Data file.

Supplementary Table 6 Multivariate Cox regression analysis of the multi-classifier system with RFS in the three sets

	Training set (n=382)		Independent validation set (n=207)		TCGA set (n=204)	
	**HR (95%CI)**	**p value**	**HR (95% CI)**	**p value**	**HR (95% CI)**	**p value**
Sex (woman vs man)	1.26 (0.72-2.21)	0.417	2.05 (1.05-4.00)	0.034	1.88 (0.92-3.82)	0.081
Age (≧60 years vs < 60 years)	1.30 (0.80-2.10)	0.283	1.17 (0.65-2.11)	0.591	0.61 (0.31-1.20)	0.152
Grade (4 vs 3 vs 2 vs 1)	1.20 (0.79-1.82)	0.399	1.56 (0.90-2.69)	0.110	1.38 (0.73-2.62)	0.319
Pathologic stage (III vs II vs I)	1.52 (0.89-2.60)	0.126	0.79 (0.39-1.57)	0.496	3.57 (1.64-7.79)	0.001
Multi-classifier system (High risk vs Low risk)	9.01 (3.95-20.55)	<0.001	13.78 (4.75-39.95)	<0.001	5.68 (1.93-16.73)	0.002

TCGA=The Cancer Genome Atlas; HR=hazard ratio; CI=confidence interval. HRs, 95% CIs and two-sided P values were calculated using the Cox proportional hazards model. Source data are provided as a Source Data file.

Supplementary Table 7 Multivariate Cox regression analysis of the multi-classifier system with OS in the three sets

	Training set (n=382)		Independent validation set (n=207)		TCGA set (n=204)	
	**HR (95%CI)**	**p value**	**HR (95% CI)**	**p value**	**HR (95% CI)**	**p value**
Sex (woman vs man)	1.17 (0.67-2.06)	0.585	1.44 (0.71-2.91)	0.308	1.34 (0.52-3.46)	0.543
Age (≧60 years vs < 60 years)	1.31 (0.82-2.11)	0.256	1.37 (0.75-2.50)	0.312	0.93 (0.40-2.15)	0.863
Grade (4 vs 3 vs 2 vs 1)	0.92 (0.62-1.39)	0.706	1.62 (0.92-2.88)	0.097	1.07 (0.50-2.29)	0.871
Pathologic stage (III vs II vs I)	1.59 (0.93-2.70)	0.088	0.69 (0.33-1.44)	0.320	5.04 (1.76-14.40)	0.003
Multi-classifier system (High risk vs Low risk)	8.51 (3.90-18.55)	<0.001	11.11 (3.85-32.05)	<0.001	5.08 (1.13-22.79)	0.034

TCGA=The Cancer Genome Atlas; HR=hazard ratio; CI=confidence interval. HRs, 95% CIs and two-sided P values were calculated using the Cox proportional hazards model. Source data are provided as a Source Data file.
