# Supplementary material for: A multi-classifier system integrated by clinico-histology-genomic analysis for predicting recurrence of papillary renal cell carcinoma
Source: Nat Commun. 2024 Jul 23;15:6215. doi: 10.1038/s41467-024-50369-y (PMC11266571; doi:10.1038/s41467-024-50369-y)
Supplement: Supplementary file 3 — Reporting Summary [file 41467_2024_50369_MOESM3_ESM.pdf]

nature portfolio

Corresponding author(s): Jun-Hang Luo

Last updated by author(s): May 30, 2024

Reporting Summary

Nature Portfolio wishes to improve the reproducibility of the work that we publish. This form provides structure for consistency and transparency in reporting. For further information on Nature Portfolio policies, see our Editorial Policies and the Editorial Policy Checklist.

Statistics

For all statistical analyses, confirm that the following items are present in the figure legend, table legend, main text, or Methods section.

n/a	Confirmed	
☐	☒	The exact sample size (*n*) for each experimental group/condition, given as a discrete number and unit of measurement
☐	☒	A statement on whether measurements were taken from distinct samples or whether the same sample was measured repeatedly
☐	☒	The statistical test(s) used AND whether they are one- or two-sided<br>*Only common tests should be described solely by name; describe more complex techniques in the Methods section.*
☐	☒	A description of all covariates tested
☐	☒	A description of any assumptions or corrections, such as tests of normality and adjustment for multiple comparisons
☐	☒	A full description of the statistical parameters including central tendency (e.g. means) or other basic estimates (e.g. regression coefficient) AND variation (e.g. standard deviation) or associated estimates of uncertainty (e.g. confidence intervals)
☐	☒	For null hypothesis testing, the test statistic (e.g. *F*, *t*, *r*) with confidence intervals, effect sizes, degrees of freedom and *P* value noted<br>*Give P values as exact values whenever suitable.*
☒	☐	For Bayesian analysis, information on the choice of priors and Markov chain Monte Carlo settings
☒	☐	For hierarchical and complex designs, identification of the appropriate level for tests and full reporting of outcomes
☒	☐	Estimates of effect sizes (e.g. Cohen's *d*, Pearson's *r*), indicating how they were calculated

Our web collection on statistics for biologists contains articles on many of the points above.

Software and code

Policy information about availability of computer code

Data collection	For the training and independent validation sets, RNA expression data was collected using RNA sequencing and qRT-PCR. Whole-slide images in SVS file format were created for each representative H&E slide using a KF-PRO-020 scanner (KFBIO, Ningbo, China). For the TCGA set, data and images were downloaded from the Genomic Data Commons portal (https://portal.gdc.cancer.gov/).
Data analysis	Quality control and pre-processing of FASTQ files were done using fastp to obtain the clean reads (clean data). Obtained RNA-seq paired-end clean data were then analyzed using Hisat2, Samtools 1.9, Stringtie 1.3.5, and DESeq2. After the alignment, the generated SAM files were sorted to BAM files using Samtools 1.9 (http://samtools.sourceforge.net). Subsequently, Stringtie 1.3.5 was used to assemble the transcripts using BAM files as inputs. We then used Stringtie and its prepDE.py to generate raw read count matrices for genes and transcripts. The genes were annotated by GENCODE version 22.Differential expression analysis was performed using the DESeq2 package in R software.The source code for the deep learning model is available online (https://github.com/guichengpeng1/WSI-based-deep-learning-classifier-in-papillary-renal-cell-carcinoma). All statistical tests were performed with R software version 4.1.0 (R Foundation for Statistical Computing, Vienna, Austria).

For manuscripts utilizing custom algorithms or software that are central to the research but not yet described in published literature, software must be made available to editors and reviewers. We strongly encourage code deposition in a community repository (e.g. GitHub). See the Nature Portfolio guidelines for submitting code & software for further information.

Data

Policy information about availability of data

All manuscripts must include a data availability statement. This statement should provide the following information, where applicable:

- Accession codes, unique identifiers, or web links for publicly available datasets
- A description of any restrictions on data availability
- For clinical datasets or third party data, please ensure that the statement adheres to our policy

The Raw-sequencing data generated in this study have been deposited in the Gene Expression Omnibus database under accession code GSE180777 [https://www.ncbi.nlm.nih.gov/geo/query/acc.cgi?acc=GSE180777]. All whole-slide images and patient data from the TCGA cohort used in this study are available from the Genomic Data Commons Data Portal [https://portal.gdc.cancer.gov/]. Source data are provided with this paper. The remaining data in this study will be made available to interested research partners upon reasonable request to the lead contact (J-HL) or the Institute of Precision Medicine, First Affiliated Hospital, Sun Yat-sen University, Guangzhou, 510080, China. Access to the data requires a data transfer agreement, approved by the legal departments of the requesting researcher and by all legal departments of the institutions that provided data for the study, and an ethics clearance.
The source code is available online (https://github.com/guichengpeng1/WSI-based-deep-learning-classifier-in-papillary-renal-cell-carcinoma).

Research involving human participants, their data, or biological material

Policy information about studies with human participants or human data. See also policy information about sex, gender (identity/presentation), and sexual orientation and race, ethnicity and racism.

Reporting on sex and gender	Sex was considered as an important variable in the study design. The sex information of patients with pRCC were retrospectively collected through the medical record.
Reporting on race, ethnicity, or other socially relevant groupings	The race, ethnicity or other information of patients with pRCC were all retrospectively collected through the medical record.
Population characteristics	All cases were pathologically confirmed as pRCC. Detailed information can be found in Table 1.
Recruitment	The training set included patients from the First Affiliated Hospital and Cancer Center of Sun Yat-sen University and patients from Renji Hospital of Shanghai Jiao Tong University. The independent validation set included cases from the Peking University First Hospital and the Affiliated Yantai Yuhuangding Hospital of Qingdao University. The TCGA set included patients from the TCGA dataset (TCGA-KIRP).
Ethics oversight	This study was approved by the Institutional Review Boards of the First Affiliated Hospital of Sun Yat-sen University, Sun Yat-sen University Cancer Center, Renji Hospital of Shanghai Jiao Tong University, Peking University First Hospital, and Affiliated Yantai Yuhuangding Hospital of Qingdao University. The informed consent was waived because patients were not directly recruited for this study.

Note that full information on the approval of the study protocol must also be provided in the manuscript.

Field-specific reporting

Please select the one below that is the best fit for your research. If you are not sure, read the appropriate sections before making your selection.

[x] Life sciences   [ ] Behavioural & social sciences   [ ] Ecological, evolutionary & environmental sciences

For a reference copy of the document with all sections, see nature.com/documents/nr-reporting-summary-flat.pdf

Life sciences study design

All studies must disclose on these points even when the disclosure is negative.

Sample size	A total of 793 patients with pRCC were included in this study. The training set comprised 382 pRCC cases, providing a robust foundation for the development of a multi-classifier system. Furthermore, the study featured two external validation cohorts, consisting of 207 and 204 cases, respectively.
Data exclusions	Inclusion criteria were patients with sporadic unilateral pRCC, stage I–III, who underwent resection without neoadjuvant therapy or adjuvant therapy, and for whom clinicopathological characteristics, follow-up information, and fixed tumor tissue were available. Patients not meeting these criteria were excluded from the study.
Replication	After the development of the multi-classifier system based on the Training set with 382 cases, the independent validation sets with 207 cases and the TCGA set with 207 cases were used to externally verify the reproducibility of the predictive performance of the multi-classifier system. All attempts at replication were successful.
Randomization	The Training, independent validation and the TCGA sets were consecutive patients, therefore, randomization was not needed. During the development of the WSI-based classifier, the developing cohort was randomly split into three sets—a discovery set, a tuning set , and a holdout internal test set.

Blinding	Pathologists were blinded to clinical characteristics and prognosis of the pRCC patients when selecting the regions of interest for features extraction.

Reporting for specific materials, systems and methods

We require information from authors about some types of materials, experimental systems and methods used in many studies. Here, indicate whether each material, system or method listed is relevant to your study. If you are not sure if a list item applies to your research, read the appropriate section before selecting a response.

Materials & experimental systems

n/a	Involved in the study
☒	☐ Antibodies
☒	☐ Eukaryotic cell lines
☒	☐ Palaeontology and archaeology
☒	☐ Animals and other organisms
☐	☒ Clinical data
☒	☐ Dual use research of concern
☒	☐ Plants

Methods

n/a	Involved in the study
☒	☐ ChIP-seq
☒	☐ Flow cytometry
☒	☐ MRI-based neuroimaging

Clinical data

Policy information about clinical studies

All manuscripts should comply with the ICMJEguidelines for publication of clinical research and a completedCONSORT checklist must be included with all submissions.

Clinical trial registration	This study is a retrospective study.
Study protocol	See methods.
Data collection	For the Training set and the independent validation set, baseline, demographic, treatment, efficacy, follow-up, and other relevant data were collected at each center during each patients' visits.<br>For the TCGA set, the data was downloaded from the Genomic Data Commons portal (https://portal.gdc.cancer.gov/).
Outcomes	The primary outcome was recurrence-free survival defined as the time from surgery to local recurrence and distant metastasis. The secondary outcome was overall survival, defined as the time from surgery to death from any cause.

Plants

Seed stocks	*Report on the source of all seed stocks or other plant material used. If applicable, state the seed stock centre and catalogue number. If plant specimens were collected from the field, describe the collection location, date and sampling procedures.*
Novel plant genotypes	*Describe the methods by which all novel plant genotypes were produced. This includes those generated by transgenic approaches, gene editing, chemical/radiation-based mutagenesis and hybridization. For transgenic lines, describe the transformation method, the number of independent lines analyzed and the generation upon which experiments were performed. For gene-edited lines, describe the editor used, the endogenous sequence targeted for editing, the targeting guide RNA sequence (if applicable) and how the editor was applied.*
Authentication	*Describe any authentication procedures for each seed stock used or novel genotype generated. Describe any experiments used to assess the effect of a mutation and, where applicable, how potential secondary effects (e.g. second site T-DNA insertions, mosiacism, off-target gene editing) were examined.*
